# Adolescent maternal health services utilization and associated barriers in Sub-Saharan Africa: A comprehensive systematic review and meta-analysis before and during the sustainable development goals

**DOI:** 10.1016/j.heliyon.2024.e35629

**Published:** 2024-08-03

**Authors:** Tadesse Tolossa, Lisa Gold, Merga Dheresa, Ebisa Turi, Yordanos Gizachew Yeshitila, Julie Abimanyi-Ochom

**Affiliations:** a Department of Public Health, Institutes of Health Sciences, Wollega University, Nekemte, Ethiopia; b Deakin University, Deakin Health Economics, School of Health and Social Development, Institute for Health Transformation, Faculty of Health, Geelong, 3220, Australia; c Haramaya University, College of Health and Medical Sciences, Department of Nursing and Midwifery, Harar, Ethiopia; d School of Nursing, College of Medicine and Health Science, Arba Minch University, Arba Minch, Ethiopia; e Intergenerational Health, Murdoch Children’s Research Institute, Parkville, Victoria, Australia

**Keywords:** Adolescent, Adolescent girls, Teenagers, Maternal health, Antenatal care, Prenatal care, Skilled birth attendant, Skilled delivery, Postnatal care, Pregnancy, Meta-analysis, Systematic review, SSA

## Abstract

**Introduction:**

Effective and adequate maternal health service utilization is critical for improving maternal and newborn health, reducing maternal and perinatal mortality, and important to achieve global sustainable development goals (SDGs). The purpose of this systematic review was to assess adolescent maternal health service utilization and its barriers before and during SDG era in Sub-Saharan Africa (SSA).

**Methods:**

Systematic review of published articles, sourced from multiple electronic databases such as Medline, PubMed, Scopus, Embase, CINAHL, PsycINFO, Web of Science, African Journal Online (AJOL) and Google Scholar were conducted up to January 2024. Assessment of risk of bias in the individual studies were undertaken using the Johanna Briggs Institute (JBI) quality assessment tool. The maternal health service utilization of adolescent women was compared before and after adoption of SDGs. Barriers of maternal health service utilization was synthesized using Andersen's health-seeking model. Meta-analysis was carried out using the STATA version 17 software.

**Results:**

Thirty-eight studies from 15 SSA countries were included in the review. Before adoption of SDGs, 38.2 % (95 % CI: 28.5 %, 47.9 %) adolescents utilized full antenatal care (ANC) and 44.9 % (95%CI: 26.2, 63.6 %) were attended by skilled birth attendants (SBA). During SDGs, 42.6 % (95 % CI: 32.4 %, 52.8 %) of adolescents utilized full ANC and 53.0 % (95 % CI: 40.6 %, 65.5 %) were attended by SBAs. Furthermore, this review found that adolescent women's utilization of maternal health services is influenced by various barriers, including predisposing, enabling, need, and contextual factors.

**Conclusions:**

There was a modest rise in the utilization of ANC services and SBA from the pre-SDG era to the SDG era. However, the level of maternal health service utilization by adolescent women remains low, with significant disparities across SSA regions and multiple barriers to access services. These findings indicate the importance of developing context-specific interventions that target adolescent women to achieve SDG3 by the year 2030.

## Highlights

• To achieve United Nation's SDG3, improving adolescent maternal health is crucial.
• This study aims to conduct a systematic review of adolescent maternal service utilization before and after the adoption of SDGs in SSA.
• Modest progress has been reported in the utilization of maternal healthcare from the pre-SDG to SDG with significant regional disparities.
• Age and context-specific interventions that address the unique needs of adolescents are crucial for achieving SDG3 by the year 2030.

## 1 Introduction

Adolescent pregnancy, occurring in women aged 10 to 19, remains a global concern [1]. While the global adolescent birth rate has shown a decline from 64.5 births per 1000 women in 2000 to 41.3 births per 1000 women in 2023, it remains high in Sub-Saharan Africa (SSA) region, with 97.9 births per 1000 women in 2023 [2]. Adolescent pregnancy often has negative physiological and social consequences [3,4]. The maternal mortality rate among adolescent women was 260 per 100,000 live births, surpassing that of women aged over 19 years, reported as 190 per 100,000 live births in 2014 [5]. SSA has the world's highest maternal mortality rates, disproportionately affecting adolescent women [6].

In SSA, limited access to education, healthcare, reproductive health information, economic opportunities, and cultural norms make adolescent women vulnerable to early, unplanned pregnancies [3,7]. These pregnancies pose health risks, including maternal mortality and adverse fetal outcomes like premature birth, low birth weight, and neonatal death [3,8].

In 2015, the United Nations set Sustainable Development Goals (SDGs), including SDG3, aiming to reduce maternal mortality to 70 deaths per 100,000 live births by 2030 [9]. Despite the ambitious targets set by SDG3.1, the maternal mortality rate has remained stagnant following the implementation of the SDGs [10]. As of 2023, the global maternal mortality rate stands at 223 deaths per 100,000 live births, which is considerably far from the target set by SDGs [11]. This stagnation could be due to resources limitations [12], lack of priorities due to conflicts and the COVID-19 pandemic [13], regional health system weaknesses [14], and policy gaps [12]. Access to essential maternal health services, such as antenatal care (ANC), skilled delivery assistance, and postnatal care (PNC) is critical for preventing maternal and child deaths, especially among adolescents [9]. Although adolescent pregnancy and maternal mortality rates are highest in SSA globally [6], adolescent maternal health service utilization remains low in this region [15]. A review conducted in SSA revealed that many adolescent women lack access to maternal healthcare due to various factors, including individual, interpersonal, community, and contextual barriers [16]. Utilization rates vary widely across SSA countries, ranging from 12.6 % in Ethiopia [17] to 61.7 % in Kenya [18].

To achieve the United Nations' SDG3 target, it is crucial to focus on adolescent maternal health, as their health outcomes will have a significant impact on progress toward the SDGs [19]. Investing in the maternal health of adolescent women can have long-term benefits, breaking poverty cycles, enhancing education, and ensuring healthier generations [20]. Revisiting maternal service utilization is essential to understand evolving challenges, assess progress, and identify gaps, prioritize adolescent rights and needs, and develop targeted intervention, especially during the pre-to-post-SDG transition. Barriers of maternal service utilization identified by Andersen's health-seeking behavioral model which include predisposing, enabling, need, and contextual factors [21]. Predisposing factors, such as demographics and pre-existing knowledge, play a significant role in adolescents' healthcare preferences. Enabling factors, related to resources and economies, are particularly relevant in SSA due to its poor economic status and healthcare infrastructure. Need factors including immediate health outcomes, affects adolescents' decisions to seek and access care. Contextual factors consisting of environmental, policy, and health system factors, affecting adolescents' service availability, acceptability, accessibility and affordability [21,22].

To date, there has been no synthesis of available data on adolescent maternal service utilization in SSA during the SDG era. Therefore, this study aims to conduct a comprehensive systematic review of adolescent maternal health service utilization before and after the adoption of SDGs in SSA. This study is crucial as it informs policy makers in prioritizing adolescent unique challenges in accessing maternal health services, guides targeted interventions, and helps in improving health outcomes of adolescent women.

## 2 Methods and materials

### 2.1 Registration

The study protocol has been registered in the International Prospective Register of Systematic Reviews (PROSPERO), with the registration ID CRD42022370207 [23].

### 2.2 Search strategy

Using the Preferred Reporting Items for Systematic Reviews and Meta-Analyses (PRISMA) criteria, the systematic review and meta-analysis were reported [24]. A preliminary search was undertaken to check for the presence of similar systematic reviews and meta-analyses that have been published on the same topic to avoid repetition and to ensure that we had enough articles to conduct a current systematic review. All published studies were searched thoroughly using nine electronic databases: Scopus, Medline, Embase, PubMed, Web of Science, CINAHL, Psycinfo, AJOL and Google Scholar (S1 file). Unpublished studies were sought from the library catalogues of different Universities in SSA. Key concept terms such as adolescent, adolescent girls, teenagers, maternal health, antenatal care, prenatal care, skilled birth attendant, skilled delivery, postnatal care, pregnancy, meta-analysis, systematic review, SSA were developed and modified for each database. In searching different databases, search terms were combined based on different search tools (truncation, wildcards, search phrases and Boolean operators). Articles accessed from both published and unpublished data sources were compiled in Endnote version 20 reference management software [25]. After duplicate articles were removed, the articles were exported to Covidence software for further screening [26]. References of included studies were accessed and reviewed for further inclusion. The search was conducted from September 5, 2022, to January 1, 2024.

### 2.3 Selection of articles and eligibility criteria

The overall search strategy and eligibility criteria were developed according to the Co–Co-Pop framework for observational studies [27].

• Condition- All articles conducted on antenatal care (ANC), skilled birth attendants (SBA) and postnatal care (PNC) utilization and their barriers were reviewed.
• Context- Studies conducted in SSA countries were considered for the review (S1 file).
• Population- Studies conducted among adolescent women.
• Study design: All observational study designs including cross-sectional (both qualitative and quantitative), case‒control and cohort study designs were included in the review.
• Language: Articles published in English were eligible for the review.
• Publication: Published and unpublished studies were considered.
• Time: All studies published on adolescent maternal service utilization between 2000 and January 2024 were considered for review.
• Sample size: No restrictions were placed on sample size for eligibility criteria.

Gray literature (e.g., conference papers, government reports, newsletters and proceedings), preprints, abstracts, editorials, commentary reports, and nonhuman studies were excluded. The primary author attempted contact for articles with incomplete data; those inaccessible after contacting the principal investigator were excluded.

### 2.4 Outcome measurement

This study has two main outcomes. The first outcome was to assess the utilization of maternal health services by adolescent women in SSA. Maternal health service utilization includes ANC, SBA, and PNC. ANC utilization is categorized as "utilized full ANC follow-up" (four or more visits) or "low utilization" (at least one but <4 visits). SBA refers to skilled health professionals assisting during childbirth. PNC utilization measures care received within six weeks post-delivery [28,29]. The second outcome of this study was the determinants and barriers associated with maternal health service utilization among adolescent women in SSA.

### 2.5 Methodological quality and data extraction

Assessment of risk of bias in the individual studies were undertaken using the Johanna Briggs Institute (JBI) quality assessment tool for observational studies [30]. The tool has 10 items for qualitative studies and 8 items for quantitative studies. The response of the tool is “yes”, “no” or “unclear” where “yes” shows that the quality is met. Studies that scored ≥4 “yes” response were included in the review [31]. Two reviewers (TTD, ET) assessed article inclusion through a four-step process: selection, title/abstract screening, full-text review, and quality assessment. Any disagreements that arose between the two reviewers were resolved by involving a third reviewer (JAO). Data were extracted by two data extractors (TTD and YY) using a standardized data extraction checklist on Microsoft Excel [32]. For the first outcome (maternal health service utilization), data included author, year, country, study design, sample size, outcome measurement, data collection method, sampling technique, and number of women utilized service. The second outcome (determinants) involved creating 2 × 2 tables to compute log odds ratios for studies examining maternal health service utilization determinants. Studies addressing maternal health service utilization barriers were thematically synthesized using Andersen's health-seeking model, categorized as predisposing, enabling, need, and contextual barriers [21].

### 2.6 Statistical analysis

Data for quantitative studies were retrieved in Microsoft Excel spreadsheet format and imported into STATA version 17 statistical software for analysis [33]. The prevalence, logarithm, and standard error of the odds ratio (OR) for each included study were generated using the “generate” command in STATA. The reported service use from each included study and the pooled result across studies were presented in the form of a forest plot. The presence of heterogeneity among the included studies was assessed by Cochran's Q test (reported as the P-value) and inverse variance index (I^2^) [34]. A random-effects model was computed to estimate the pooled maternal health service utilization. Subgroup analysis was conducted to compare maternal health service utilization in two time periods: “pre-SDGs” (2000–2015), also known as the period of the Millennium Development Goals (MDGs) and “SDGs” (2016 onwards), the period after the adoption of the SDGs. Subgroup analysis was also conducted to identify the source of heterogeneity, specifically to explore differences between studies conducted in Western, Eastern and Southern regions of SSA [35] and between different study types (primary versus secondary data analyses and quantitative versus mixed method studies). We considered maternal health service utilization before and after the adoption of the SDGs using the study period rather than the year of publication to account for potential time difference between data collection and publication. Funnel plot and sensitivity analysis was performed to see the publication bias and the effect of single study on overall studies respectively.

## 3 Results

### 3.1 Search results

**Fig. 1:**
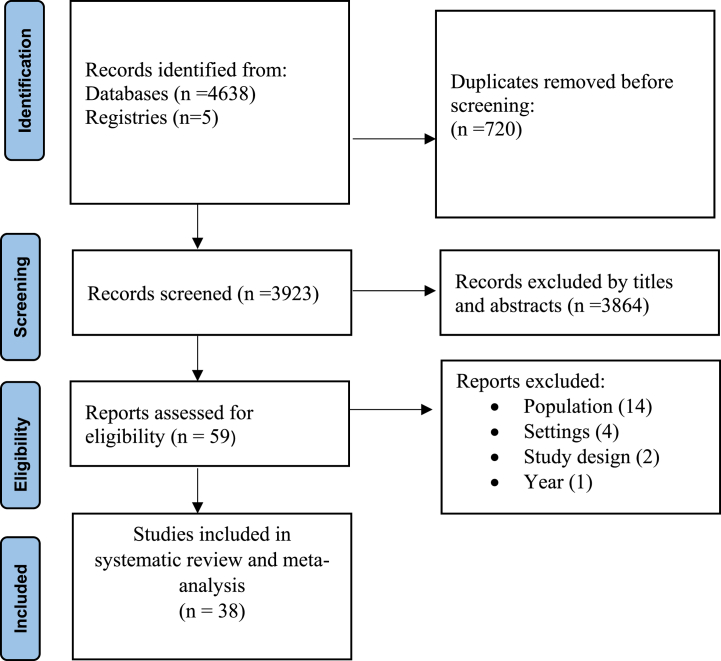
PRISMA flow diagram of systematic review and meta-analysis.

A total of 4643 studies were identified from the search strategy. After removal of duplicates, the remaining 3923 articles were screened and 3864 excluded after reading titles and abstracts. Full texts of the remaining 59 articles were assessed, and 38 studies included in the final systematic review and meta-analysis (Fig. 1).

### 3.2 Characteristics of the included studies

Of the 38 studies included in the review, 27 studies were published after 2015 [[36], [37], [38], [39], [40], [41], [42], [43], [44], [45], [46], [47], [48], [49], [50], [51], [52], [53], [54], [55], [56], [57], [58], [59], [60]]. However, when considering the timing of data collection in included studies, 20 studies were conducted during the pre-SDG era [17,37,40,41,44,46,57,59,[61], [62], [63], [64], [65], [66], [67], [68], [69], [70], [71], [72]], and 18 studies were conducted during the era of SDG [38,39,42,43,45,[47], [48], [49], [50], [51], [52], [53], [54], [55], [56],^58^,^60^]. The 38 studies included a total of 58097 adolescent women. Of the 38 included studies, more than half (17) were conducted using a quantitative cross-sectional study design [17,[36], [37], [38],^40^,^41^,^43^,^47^,^49^,^56^,^57^,^62^,^63^,[68], [69], [70],^72^], 14 studies were conducted using a qualitative study design [39,42,44,45,48,54,55,58,60,61,[65], [66], [67],^71^], and the remaining seven [46,[50], [51], [52], [53],^59^,^64^] were conducted with a mixed method. Studies from 15 SSA countries and 4 multicounty studies [43,48,49,57] were included in the review. The largest number of studies (eight) were conducted in Nigeria [[36], [37], [38],[50], [51], [52],^56^,^69^], followed by Uganda [39,55,61,62,71] and South Africa [45,46,58,65,66]. Three studies were from Kenya [40,53,73], two each from Malawi [44,70] and two Zimbabwe [63,64] and one each from Ethiopia [17], Zambia [42], Namibia [60], Tanzania [54], Niger [70], Mali [72], Guinea [47], Lesotho [67], and Ghana [59]. Two-thirds (65 %) of the studies were conducted at the community level [17,[36], [37], [38], [39],^41^,^43^,[47], [48], [49], [50], [51], [52],^54^,^56^,^57^,^59^,^62^,[68], [69], [70]], and the remaining eleven studies (35 %) were facility-based studies [42,[44], [45], [46],^53^,^55^,^58^,^60^,^61^,[63], [64], [65], [66], [67],^71^] (Table 1).

**Table 1:** Summary of studies included in the systematic review and meta-analysis.

S.N	Author	Year of publ.	Country	Study design	Data collection period	Setting	Source of data	Sampling technique	Types of maternal service utilization and outcome measurement	Risk of bias assessment
1	Alemayehu T et al. [17]	2010	Ethiopia	Cross-sectional	Pre-SDG	Community based	Secondary data	Multistage sampling and snowball	ANC-Attending ANC at least 4 visits	7
									SBA- Delivery conducted by HPs in HF	
2	Apolot R et al. [39]	2020	Uganda	Qualitative study	SDG	Community based	Primary data	Purposive Sampling	ANC, SBA and PNC- Challenges faced by adolescents during pregnancy, delivery, and the post-natal period	8
3	Atuyambe L et al. [61]	2009	Uganda	Qualitative study	Pre-SDG	Facility based	Primary data	Purposive Sampling	ANC- Explore adolescent health seeking behaviour during pregnancy	6
4	Hackett K et al. [48]	2019	Tanzania and Ghana	Qualitative study	SDG	Community based	Primary data	Purposive Sampling	ANC- Adolescent girls experience of ANC utilization	7
5	Rukundo G et al. [71]	2015	Uganda	Qualitative study	Pre-SDG	Facility based	primary data	Purposive Sampling	ANC- Availability, accessibility, and utilization of teenager friendly antenatal services	9
6	Rai RK et al. [69]	2012	Nigeria	Cross-sectional	Pre-SDG	Community based	Secondary data	Equal probability systematic	ANC- Attending ANC at least 4 visits.	6
									SBA- Delivery conducted by HPs in HF.	
									PNC- Postnatal follow-up within 2 months of delivery	
7	Shatilwe et al. [60]	2022	Namibia	Qualitative study	SDG	Facility based	Primary data	–	ANC- Explore accessibility and utilization of service during pregnancy	7
8	Olakunde et al. [56]	2019	Nigeria	Cross-sectional	SDG	Community based	Secondary data	Cluster sampling	SBA- Delivery conducted by HPs in HF	6
9	Samuel N et al. [55]	2022	Uganda	Qualitative study	SDG	Facility based	Primary data	Convenience sampling	ANC- Explores barriers of maternal health services during pregnancy	9
10	Duggan R et al. [65]	2012	South Africa	Qualitative study	Pre-SDG	Facility based	Primary data	Purposive Sampling	ANC and PNC- Adolescents perceptions and expectations of maternity services	7
11	Owolabi O et al. [57]	2017	Multi-country	Cross-sectional	Pre-SDG	Community based	Secondary data	Multistage stratified sampling	ANC- Attending ANC at least 4 visits.	7
12	Shamaternal service utilization D et al. [59]	2018	Ghana	Mixed study	Pre-SDG	Community based	Primary data	Purposive Sampling	ANC- Attending ANC at least 4 visits.	5
									SBA- Delivery conducted by HPs in HF	
13	Mweteni W et al. [54]	2021	Tanzania	Qualitative study	SDG	Community based	Primary data	Purposive Sampling	ANC- Pregnant adolescents' barriers and facilitators to accessing ANC	10
14	Singh PK et al. [72]	2013	Mali	Cross-sectional	Pre-SDG	Community based	Secondary data	Stratified, two stage cluster sampling	ANC- Attending ANC at least 4 visits.	6
									SBA-Delivery assisted by a doctor, nurse, or midwife.	
									PNC- Postnatal follow-up within 2 months of delivery	
15	Mekwunyei L et al. [50]	2022	Nigeria	Mixed study	SDG	Community based	Primary data	Multistage sampling and snowball	ANC- ANC visit appropriate with their gestational age	6
16	Akinyemi A et al. [36]	2021	Nigeria	Cross-sectional	SDG	Community based	Primary data	Random sampling	ANC- Attending ANC at least 4 visits.	7
17	Rai RK et al. [70]	2014	Niger	Cross-sectional	Pre-SDG	Community based	Secondary data	Stratified two-stage cluster	ANC- Attending ANC at least 4 visits.	8
									SBA- Delivery conducted by HPs at health facility	
18	Thomas A et al. [40]	2017	Kenya	Cross-sectional	Pre-SDG	Community based	Secondary data	Two stage cluster sampling	ANC- Attending ANC at least 4 visits.	8
									SBA- Delivery assisted by HPs.	
									PNC- Care given six-week period following delivery	
19	Mulinge N et al. [53]	2017	kenya	Mixed study	SDG	Facility based	Primary data	Multistage random sampling	ANC- Attending ANC at least 4 visits	7
20	CN Chaibva et al. [63]	2009	Zimbabwe	Cross-sectional	Pre-SDG	Facility based	Primary data	Purposive, nonprobability sampling	ANC- Assessing regular care and monitoring given to a woman during pregnancy	9
21	GrovoguiI F et al. [47]	2022	Guinea	Cross-sectional	SDG	Community based	Secondary data	Multilevel cluster sampling	ANC- Attending ANC at least 4 visits.	7
									SBA- Care given by HPs at government health facility	
22	Chikalipo et al. [44]	2018	Malawi	Qualitative study	Pre-SDG	Facility based	Primary data	Purposive sampling	ANC- Attending ANC at least 4 visits.	8
									PNC-Postnatal care within 42 days of delivery	
23	Erasmus M et al. [45]	2020	South Africa	Qualitative study	SDG	Facility based	Primary data	Purposive sampling	ANC- Barriers to accessing maternal health care of adolescent women	8
24	Rai RK et al. [70]	2014	Malawi	Cross-sectional	Pre-SDG	Community based	Secondary data	Stratified two-stage cluster design	ANC- At least four antenatal care visits, PNC- Care within 42 days of delivery	8
25	Atuyambe Let al. [62]	2008	Uganda	Cross-sectional	Pre-SDG	Community based	Primary data	Multistage and cluster sampling	ANC- Attending ANC at least 4 visits.	8
									SBA- Delivery attended at health facility by HPs	
26	Alex-Ojei et al. [37]	2020	Nigeria	Cross-sectional	Pre-SDG	Community based	Secondary data	Multistage cluster sampling	SBA-Delivery assisted by a doctor, nurse, or midwife	8
27	Iacoella [49]	2019	Multi-country	Cross-sectional	SDG	Community based	Secondary data	Multistage cluster sampling	ANC- Attending ANC at least 4 visits.	8
									PNC- Postnatal care within 2 months of delivery	
28	C.A. Alex et al. [38]	2021	Nigeria	Cross-sectional	SDG	Community based	Secondary data	Multistage cluster sampling	ANC- Attending ANC at least 4 visits.	7
									SBA- Delivery attended at health facility by HPs	
29	Bwalya et al. [42]	2018	Zambia	Qualitative study	SDG	Facility based	Primary data	Purposive Sampling	ANC- Experience of adolescent women service utilization	8
30	Carvajal et al. [43]	2020	Multi-country	Cross-sectional	SDG	Community based	Secondary data	Multistage cluster sampling	ANC- Attending ANC at least 4 visits.	6
									SBA- Delivery attended at health facility by HPs	
31	Banke-T et al. [41]	2018	Kenya	Cross-sectional	Pre-SDG	Community based	Secondary data	Multistage cluster sampling	ANC- Attending ANC at least 4 visits.	8
									SBA- Delivery by skilled HPs in health facility	
									PNC- Postnatal care within 2 months of delivery	
32	Govendera T et al. [46]	2018	South Africa	Mixed study	Pre-SDG	Setting	Primary data	Convenience sampling	ANC- Attending ANC at least 4 visits	8
33	James S et al. [66]	2012	South Africa	Qualitative study	Pre-SDG	Community based	Primary data	Purposive sampling	ANC- Experience of attendance of the ANC clinic by adolescent women	7
34	Phafoli Sh et al. [67]	2014	Lesotho	Qualitative study	Pre-SDG	Community based	Primary data	Purposive sampling	ANC- Explore the reason for delayed ANC initiation	8
35	Sewpaul R et al. [58]	2021	South Africa	Qualitative study	SDG	Facility based	Primary data	Purposive sampling	ANC- Explored experiences of pregnant adolescents' treatment by HCWs	6
36	Michael T et al. [51]	2023	Nigeria	Mixed study	SDG	Community based	Primary data	Multistage sampling and purposive	ANC- Attending ANC at least 4 visits	7
37	Michael T et al. [52]	2021	Nigeria	Mixed study	SDG	Facility based	Primary data	–	SBA- Delivery by skilled HPs in health facility	7
38	Chaibva C et al. [64]	2010	Zimbabwe	Mixed study	Pre-SDG	Community based	Primary data	–	ANC- Midwives' perceptions reason for delay and non-utilization of prenatal services	7

ANC- antenatal care, HF- health facility, HPs-health professionals, MDG- Millennium Development Goal, SDG- Sustainable Development Goal, IDI- In-depth interview, FDG-focus group.

### 3.3 Level of maternal health service utilization

**Fig. 2:**
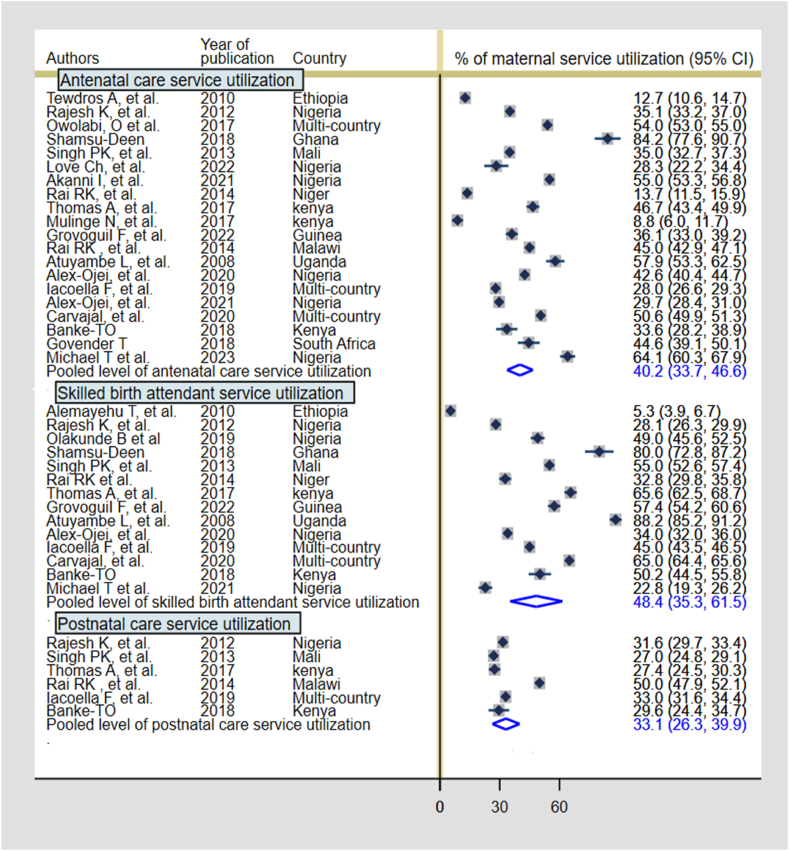
Adolescent maternal service utilization (ANC, SBA, PNC) in SSA.

Twenty studies assessed the level of ANC utilization among adolescents [17,[36], [37], [38],^40^,^41^,^43^,^46^,^47^,[49], [50], [51],^53^,^57^,^59^,^62^,[68], [69], [70],^72^], 14 studies assessed the level of SBA [17,38,40,41,43,47,49,52,56,59,62,69,70,72], and six studies reported the level of PNC utilization [40,41,49,68,69,72]. From the meta-analysis, the pooled level of utilising ≥4 ANC visits by adolescent women was 40.2 % (95 % CI: 33.7 %, 46.6 %). In SSA, the pooled level of SBA and PNC service utilization among adolescent women were 48.4 % (95 % CI: 35.3 %, 61.5 %) and 33.1 % (95 % CI: 26.3 %, 39.9 %) respectively (Fig. 2).

### 3.4 ANC and SBA utilization before and during SDG

**Fig. 3:**
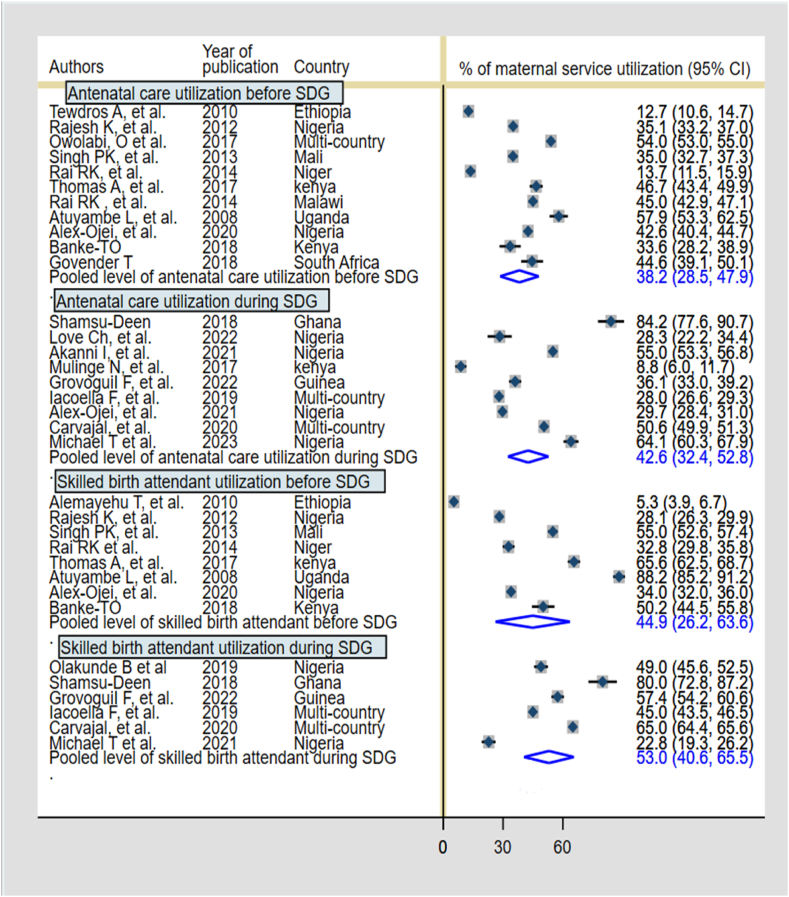
Comparison of ANC and SBA utilization by adolescent women before and during SDG in SSA.

The pooled level of ANC and SBA were compared before and after the adoption of SDGs. Accordingly, the level of ANC utilization before SDG adoption was 38.2 % (95 % CI: 28.5 %, 47.9 %) and 42.6 % (95 % CI: 32.4 %, 52.8 %) during the era of SDG. The level of SBA utilization before SDG adoption and during SDG were 44.9 % (95%CI:26.2, 63.6 %) and 53.0 % (95 % CI: 40.6 %, 65.5 %) respectively. The comparison of PNC service utilization before SDG adoption and during SDG was not conducted due to a lack of studies that reported PNC utilization during the SDG era (Fig. 3).

### 3.5 ANC and SBA utilization across SSA regions

ANC utilization varied across SSA regions, with the highest in Southern Africa at 44.5 % (95 % CI: 39.1, 50.1) and the lowest in Easten Africa at 33.5 % (95 % CI: 28.2, 38.9). SBA utilization was lowest in Eastern Africa (27.5 %, 95 % CI: 26.3, 28.9) compared to Western Africa (38.6 %, 95 % CI: 37.6, 39.5). Variability was observed in study designs, data types, and study settings (Table 2).

**Table 2:** Summary of subgroup analysis for ANC and SBA service utilization.

Subgroup		ANC utilization			SBA utilization		
		No of studies	Level of ANC (95%CI)	Study heterogeneity (I^2^ and P value)	No of studies	Level of SBA (95%CI)	Study heterogeneity (I^2^ and P value)
Study design	Cross-sectional	15	38.3 % (95 % CI:31.4, 45.2)	(99.6 %, P < 0.001)	12	47.9 % (95 % CI:33.8, 62.1)	(99.9 %, P < 0.001)
	Mixed	5	45.9 % (95 % CI: 17.5, 74.3)	(99.5 %, P < 0.001)	2	51.3 % (95 % CI: 4.7, 107.3)	(99.5 %, P < 0.001)
Source of data	Secondary	11	37.6 % (95 % CI: 30.2, 45.1)	(99.6 %, P < 0.001)	10	43.8 % (95%CI: 28.3, 59.3)	(99.9 %, P < 0.001)
	Primary	9	43.4 % (95 % CI: 27.4 %, 59.4 %)	(99.6 %, P < 0.001)	4	59.9 % (95 % CI: 27.9, 92.0)	(99.6 %, P < 0.001)
Region	Western Africa	11	43.3 % (95 % CI:34.3, 52.2)	(99.5 %, P < 0.001)	8	38.6 % (95 % CI: 37.6, 39.5)	(99.6 %, P < 0.001)
	Eastern Africa	6	34.1 % (95 % CI:17.8, 50.2)	(99.5 %, P < 0.001)	4	27.5 % (95 % CI: 26.3, 28.6)	(99.6 %, P < 0.001)
	Southern Africa	1	44.5 % (95 % CI: 39.1, 50.0)	–			

### 3.6 Methodological quality, publication bias and sensitivity analysis

All studies included in the review scored 4 and above using JBI risk of bias assessment tool. Publication bias was assessed both graphically, showing asymmetry in the funnel plot, and statistically using Egger's weighted test, which did not reveal a significant presence of publication bias (P = 0.200 for ANC and 0.359 for SBA) (S2 file). Sensitivity analysis showed no strong evidence of individual studies significantly influencing the overall results of the remaining studies.

### 3.7 Determinants and barriers of maternal health service utilization

The determinants and barriers of maternal health service utilization was assessed using Andersen's health-seeking model, categorizing them into predisposing, enabling, need, and contextual barriers, further segmented by thematic areas (Table 4). The result of quantitative meta-analysis was summarized using Table 3.

**Table 3:** Determinants of maternal service utilization among adolescent women in SSA.

Variables	Categories	OR with 95%CI	I^2^ and P value	Number studies	Sample size
Age	<18 years	ref	(75.0 %, P = 0.001)	6	9323
	18–19 years	1.36 (1.07, 1.71)			
Residence	Rural	ref	(98.6 %, P < 0.001)	6	9836
	Urban	1.44 (0.52, 3.99)			
Marital status	Single	ref	(97.6 %, P < 0.001)	5	7677
	Married	1.41 (0.43, 4.60)			
Religion	Christian	1.68 (0.87, 3.27)	(87.8 %, P < 0.001)	4	1482
	Others	ref			
Women's educational status	No formal education	ref	(98.5 %, P < 0.001)	9	17,713
	Formal education	2.02 (1.08, 3.79)			
Partner's educational status	No formal education	ref	(98.5 %, P < 0.001)	5	11,930
	Formal education	1.94 (1.80, 2.10)			
Employment	Unemployed	ref	(0.00 %, P = 0.662)	2	2031
	Employed	1.24 (1.02, 1.51)			
Wealth status	Poor	ref	(98.3 %, P < 0.001)	5	12,828
	Rich	1.67 (0.92, 3.05)			
Media exposure	No exposure	ref	(0.00 %, P = 0.544)	2	1947
	Exposure	3.93 (2.87, 5.38)			
Household head	Male	ref	(74.8 %, P = 0.046)	2	3742
	Female	1.66 (1.12, 2.47)			
Parity	One	ref	(0.00 %, P = 0.602)	2	1199
	Multiple	0.78 (0.47, 1.28)			
Pregnancy intention	Unplanned	ref	(72.8 %, P = 0.005)	5	10219
	Planned	0.97 (0.72, 1.29)

**Table 4:** Summary of the barriers and determinants of maternal service utilization among adolescent women in SSA.

Framework	Categories	Barriers
**Predisposing barriers**	Socio-demographic barriers	•Age <18 years [37,51,56,59,70], lack of education [17,36,38,40,41,47,49,51,59,69,70,72], low educational status of the family [17,36], low educational status of husband [37,38,40,41,49,59,69,72], rural residence [17,40,41,47,49,54,59,69,70,72], low birth order and interval [49,69,70,72], religious factors [47,63,68,69], unmarried marital status [36,49,50,53,59], male household head [37], ethnicity [41], lack of parents [51].
	Health knowledge and beliefs	•Lack of knowledge about ANC [45,53,54,58,59,63,64,67], lack of awareness [61,65,69], cultural mal-practices or unwritten community laws [39,54,59,61], using herbs [61]
**Enabling barriers**	Individual level barriers	•Lack of money for transportation [39,46,48,50,55,60,61], lack of income [46,51,52,54,59,60,62,63,67], lack of money for maternity wear [39], lack of autonomy [17,38,49,53,54,60,70], fear of health professionals [48], shyness and embarrassment [46,48], fear of adult women [71], fear of disclosing pregnancy [45,60,63], lack of privacy and confidentiality [55]
	Family related barriers	•Poor wealth status of family [17,[36], [37], [38],^40^,^41^,^47^,^49^,^51^,^56^,^62^,^69^,^70^], rejection by partner and not accepting paternity [39,61,62,67,71], not accepting her pregnancy by family [39,45,67], harsh treatment by family and use of abusive language (due to pregnancy before marriage and unknown husband) [39,50,54,60,62], family denied food, money, and bed [39], lack of family support [39,59,64,65,71], absence of male involvement [51]
	Community level barriers	•Stigma from community [17,42,45,48,49,60,61], fear of peers and community [39], lack of social support [48,71], society does not promote the use of PNC [69], presence of TBA in community [[50], [51], [52],^63^,^64^], lack of transport and poor infrastructure [52,60], lack of media exposure [40,41,49,54,60,69,72]
**Need factors**		•Lack of birth preparedness [39], place of delivery affect PNC [61,69,72], unplanned pregnancy [36,37,41,53,[70], [71], [72]], lack of ANC [37,47,56,69,70,72], fear of caesarean section [50], coercion and violence from friends and family, negative emotional response to pregnancy (sadness, fear, and guilt) when they are pregnant [45], fear of HIV status (since they had sex without condom) [46,64], only visiting ANC when they develop medical problem otherwise utilising TBAs (assuming ANC is only for sick) [51]
**Contextual factors**	Availability (HP and infrastructure)	•Lack of adolescent friendly services [39], lack of comprehensive services such as family planning and post-abortion care [39,60], frequent lack of drugs and stock-outs from health facility [39,48], only conducting ANC service on specific days [48], staff shortages [48,50,66], lack of obstetric equipment [48], illegal fees and high prenatal fee [48], lack of referrals and transfers system [48], lack of adolescent waiting place and delivery place [42], lack of separate ANC clinic (giving ANC service with under-five clinic) [42,46,66], health facility does not work at night [52], long waiting times [39,42,48,59,60,65], long distance [38,46,52], indirect medical cost not covered by health facilities [39], difficulty in reaching health facility [55]
	Acceptability	•HWs give priority to women who were escorted by their partners [39], lack of compassionate care [46,58,61,67], health system policy (pregnancy is not allowed in school in Tanzania) [48], negative attitude from health workers such as insulting, using inappropriate words [39,42,45,48,51,55,59,60,65,71], lack of friendly communication between health workers and adolescents [51,64,65], dismissal of pregnant women from school [67], lack of privacy and confidentiality in health facility [42,48,65,66,71]

Abbreviation.

ANC- Antenatal care.

SBA- Skilled Birth attendant.

TBAs- Traditional Birth attendants.

PNC- Postnatal care.

### 3.8 Predisposing barriers

#### 3.8.1 Socio-demographic barriers

The educational levels of mothers, husbands, and families play a significant role in maternal health service utilization of adolescent women in SSA. A meta-analysis of nine studies revealed that educated women were more likely to use maternal health care compared to those with no formal education [[36], [37], [38],^40^,^41^,^49^,^50^,^53^,^72^] (OR = 2.02, 95 % CI: 1.08, 3.78). Similarly, women with educated partners were 1.94 times more likely to utilize maternal health care compared to those with uneducated partners (OR = 1.94, 95 % CI: 1.80, 2.10), as indicated by findings from five studies [[36], [37], [38],^40^,^72^]. The influence of education extends to the family level, where adolescent women from uneducated families were less likely to use maternal health care [17,36].

The effect of age on maternal health service utilization was assessed by using six studies [37,38,41,53,59,72] the pooled findings showed that maternal health service utilization was 1.36 times higher among women aged over 18 years than younger adolescent women (OR = 1.36, 95 % CI: 1.07, 1.71). Findings from three qualitative studies supported this finding by indicating that younger women were less likely to utilize maternal health care [56,59,70].

Five quantitative studies [37,38,41,53,59] found insignificant association between marital status and maternal health service utilization. However, the result of three qualitative studies found that women who were single, divorced and separated were less likely to utilize maternal health care than married adolescent women [36,49,50]. Religious beliefs were associated with low maternal health service utilization in four studies: in Nigeria [69] and Guinea [47] women belonging to the Muslim religion had lower uptake of the maternal health service than women belonging to catholic religion while in Malawi women of catholic faith were less likely utilize maternal health care than other religions [68]. In Zimbabwe, approximately 12.5 % of adolescent women did not attend ANC follow-up due to religious influences [63].

#### 3.8.2 Health knowledge and beliefs

Maternal healthcare utilization is influenced by women's awareness of available services and cultural beliefs. In twelve studies, women with good awareness and knowledge about available services were more likely to use maternal healthcare [45,53,54,58,59,61,[63], [64], [65],^67^,^69^]. Cultural beliefs such as unwritten community laws, and using herbs inhibit the maternal health service utilization of adolescent women [39,54,59,61]. In Tanzania, local laws require pregnant women to attend ANC with their spouse, prohibiting unmarried or unaccompanied women due to denial of paternity by their partner or spouse [54]. In Uganda, retaining the placenta at home is seen as a sign of giving birth to an intelligent child. Consequently, adolescent women tend to choose home deliveries over healthcare facilities to retain access to the placenta due to this cultural belief [61]. In Ghana, it is culturally forbidden to disclose a woman's pregnancy publicly until a specific ritual is performed, potentially delaying early ANC initiation [59].

### 3.9 Enabling factors

#### 3.9.1 Individual related factors

Fourteen studies assessed the association between cost and maternal health service utilization of adolescent women [37,39,46,48,50,54,55,[59], [60], [61], [62], [63], [64],^67^]. Of the total 14 studies, ten studies indicated the effect of direct medical cost on maternal health service utilization [37,39,46,48,50,[59], [60], [61],^63^,^64^,^67^]. The studies revealed that adolescent women often dropout from maternal healthcare services for various financial reasons. These include a lack of money to acquire essential medications [39,48,55], insufficient financial resources in emergency situations [61], and lack of money to initiate and attend ANC service [37,46,59,63,67]. In instances where health facilities impose high prenatal fees, women choose to not utilize maternal health care [48,64].

Nine studies assessed the effect of direct non-medical cost on maternal health service utilization [37,39,46,48,50,54,55,60,61]. Eight studies revealed that adolescent women faced challenges in utilising ANC services due to the financial burden of transportation costs [37,46,48,50,54,55,60,61]. A study conducted in Uganda found that lack of money for transportation is the main reasons behind adolescent women's preference for traditional birth attendants (TBAs) over trained health professionals [61]. Other studies reported the effect of cost on maternal service utilization due to lack of money for clothes and birth preparedness [39,54,61,62] and lack of money for referral to higher health facilities during complications [61].

Eight studies explored the association between decision making autonomy of the women and maternal health service utilization. Adolescent women who participated in decision making jointly with their husband/partner were more likely to utilize maternal health care than women whose husband/partner made decisions without involving the women [17,38,49,53,54,60,70]. Individual-related enabling barriers such as fear of health professionals [48], shyness and embarrassment [46,48], fear of adult women [71], and fear of disclosing pregnancy [45,60,63] were identified as the barriers of accessing maternal health care. A meta-analysis of two studies found no significant association between maternal health service utilization and number of pregnancies (OR = 0.78, 95 % CI: 0.47, 1.28) [40,41].

#### 3.9.2 Family/partner related factors

Meta-analysis of five studies found no significant association between household wealth index and adolescent maternal health service use (OR = 1.67, 95 % CI: 0.92, 3.05) [[36], [37], [38],^41^,^72^]. However, eight qualitative studies indicated that lower wealth status in families/partners reduced maternal health care utilization compared to higher wealth families [39,50,[54], [55], [56],^59^,^63^,^69^].

In SSA, many adolescent women were not accessing maternal health care due partner's refusal to accept paternity [39,61,62,67,71] and stigma associated with premarital pregnancy [39,45,67]. This rejection is often accompanied by denial of basic necessities like food and money [39], and even harsh treatment by family members [50,54,60,62]. Additionally, the absence of family support [39,59,64,65,71], and limited male involvement in maternal care further deter maternal health service utilization [51].

#### 3.9.3 Community related factors

Six studies assessed the association between residence and maternal health service utilization. Four studies Four studies reported higher service utilization among women from areas [37,40,41,72], while two studies indicated lower service utilization [38,59]. The pooled result showed a non-significant association between residence and maternal health service utilization (OR = 1.44, 95 % CI: 0.52, 3.99). Meta-analysis on media exposure and maternal health service utilization [40,41,49,54,60,69,72] found that women exposed to media were more significantly more likely to utilize maternal health care compared to those not exposed to media (OR = 3.93, 95 % CI: 2.87, 5.38).

Eight studies addressed the relationship between TBAs and maternal health service utilization with varying findings. In Ethiopia, over 80 % of women receiving ANC at health facilities opted for home births without skilled attendants [17]. In Kenya and Zimbabwe, only 7 % [40] and 8.8 % [63] of pregnant women sought care from TBAs during pregnancy and childbirth, while in Nigeria, 32.3 % of adolescent women used TBAs [38]. Reasons for TBA preference over modern health care facilities included financial constraints for transportation, birth preparations, and medication [61], fear of stigma and discrimination from the community and friends [54,61], and unfavourable attitudes of health professionals [39,61]. Cultural practices, family history, and limited decision-making power in families also influenced TBA use [48,50,59,61].

### 3.10 Need factors

A meta-analysis of five studies found no significant association between pregnancy intention and maternal health service utilization [36,37,41,53,72] (AOR = 0.97, 95 % CI: 0.72, 1.29). However, two qualitative studies [70,71] suggest that planned pregnancies lead to higher maternal health care utilization. In eight studies examining ANC and SBA utilization, full ANC attendance increased the likelihood of facility based deliveries [37,47,51,56,[68], [69], [70],^72^]. SBA users were also more likely to access PNC in two studies [61,69]. Factors like negative emotional responses during pregnancy [45], fear of HIV due to condomless sex [46,64], and using ANC only for medical issues [51] prevented women from using ANC service.

### 3.11 Contextual factors

#### 3.11.1 Availability of health professional and infrastructure

Service availability significantly affects maternal health care utilization. In Uganda, the absence of adolescent-friendly services hinders utilization among adolescent women [39]. Studies in Uganda and Namibia, also reported reduced utilization due to the lack of comprehensive maternal care, including post-abortion care and family planning [39,60]. Additionally, conducting ANC service only on specific days [48], staff shortages [48,50,66], lack of referral system [48], absence of maternity waiting rooms [42], and the absence of separate ANC clinics for adolescent women [42,46,66] were further barriers to seeking health care. Long waiting times were found to decrease maternal health service utilization in six studies [39,42,48,59,60,65] and long distances hindered or delayed service use in three studies [38,46,52]. Poor transportation and infrastructure further reduced maternal health care utilization [55].

#### 3.11.2 Acceptability of the service

The maternal health service utilization of adolescent women in SSA was influenced by poor health system and country policies. Two studies assessed the impact of health-related policies on maternal health service utilization. A study conducted in Tanzania showed that prohibition of pregnancies during and the fear of health care services after becoming pregnant hinder utilization [48]. According to a study in Lesotho, unmarried pregnant women face expulsion from school and fear discrimination and stigma, preventing them from seeking maternal health care utilization [67]. Moreover, unfavourable attitudes of health professionals including lack of compassionate care [46,58,61,67], insults and using inappropriate language [39,42,45,48,51,55,59,60,65,71], and unfriendly communication [51,64,65] discourage women from accessing services in SSA.

## 4 Discussion

This systematic review examined the level of maternal health service utilization and associated barriers among adolescent women in SSA. The findings of the study provide strong evidence of low maternal health service utilization among adolescent women and identified predisposing, enabling, need and contextual factors that influence their maternal service utilization.

The study indicates that adolescent women's ANC service utilization in SSA increased from 38.2 % pre-SDG to 42.6 % post-SDG, showing a slight positive change. This level of ANC service utilization during the SDG era aligns with findings in India [74,75] and Nepal [76], but is lower compared to studies in Indonesia [77,78], Nepal [79] and Bangladesh [80]. Difference may be due to age variations across studies with some including women aged 20–24 which can affect utilization patterns if these older women have different utilization patterns than women under 20 years. Conversely, utilization in this review was higher than previous estimates for India [81] but this may be due to different ANC definitions, potentially underestimating utilization in the India study. Additionally, SBA utilization during childbirth rose from 44.8 % pre-SDG to 53.0 % during SDG, consistent with studies conducted in Nepal [76] and India [74]. However, it was lower than the joint WHO and UNICEF report in SSA [82], and a SSA study using a recent DHS dataset [83]. Differences may arise from the broader SSA data in previous reports, while this study focused on specific SSA regions.

The increase in ANC and SBA utilization could be attributed to increased health awareness, better healthcare infrastructure, resource allocation for maternity services, the availability of facilities and trained professionals, and SDG-driven policies aimed at enhancing maternal and child health [84]. These policies include raising the target ANC visits to eight [85], implementation national health insurance schemes [86], and free maternity policy in the region [87,88]. Community health workers have also played a pivotal role in improved service access across SSA regions [[89], [90], [91]]. The greater increase in SBA compared to ANC in SSA may be linked to persistent challenges in accessing and utilising ANC services. These challenges often require early initiation and multiple visits, posing difficulties in regions with transportation and infrastructure limitations [52,60]. Despite progress, there remains a substantial gap in improving coverage for ANC, SBA and PNC service utilization. Addressing these gaps is crucial for ensuring the comprehensive well-being of both mothers and newborns, particularly within the context of adolescent women.

Regarding the regional comparison, disparities persist in utilization of the service across various SSA regions. The observed disparities might be due to a combination of social, economic, cultural, infrastructural and policy differences in the region [92,93]. The variation in service use observed across SSA regions and individual countries suggests that closing these gaps is achievable, though additional efforts may be needed especially, in regions with the lowest current service utilization, such as Eastern Africa.

Predisposing sociodemographic barriers, such as younger age group, lack of education, and having uneducated partner/husband, decrease the likelihood of maternal health service utilization among adolescent women. These aligns with previous studies conducted in different settings [[94], [95], [96], [97], [98]]. Enhancing adolescent literacy and education is a key strategy to improve adolescents' use of maternal health care. Younger women often financially dependent on their family and partners, with limited-service utilization, experience, and lower educational status, face additional challenge compared to older women. Women with higher educational attainment are more likely to be more aware healthcare services’ value, initiate service use early, and proactively planning delivery. Furthermore, educated partners contribute positively by understanding the importance of attending health facilities for ANC or delivery services, providing financially and psychological support, and mitigating discrimination and stigma within the family. These factors collectively enhance decision-making autonomy.

This review also found that lack of media exposure, insufficient knowledge, and adherence to cultural beliefs and norms decrease the likelihood of maternal health service utilization. Exposure to media and possessing adequate knowledge empower women to understand the importance of service utilization, the significance of consulting qualified health professionals, the risks associated with not receiving essential services from health facilities and facilitate active involvement in decision making with their family and partner. These findings align with studies conducted in in India [75,97], and South-east Asia [99]. Social norms and cultural beliefs further influence maternal health service utilization particularly in SSA countries, where communities often value TBAs and prefer home births under TBAs care due to cultural norms, trust in traditional practices, accessibility, lower costs, and assumed better counselling compared to trained health workers [61]. Fear of stigma and discrimination from health professionals further encourage women to choose TBAs over seeking health services from trained health professionals [50,54]. Given the significant influence of social norms and cultural beliefs on maternal health service utilization in SSA region, it is crucial to consider community perspectives and incorporate TBAs into policy design. Developing context-specific recommendations is essential to improve maternal healthcare outcomes.

High out-of-pocket expenses including consultation fees, medications, and transportation costs discourage women from seeking maternal health care. This aligns with studies in LMICs [100], India [101,102], and Myanmar [103]. While several SSA countries introduced free maternity policies to alleviate financial barriers, challenges such as insufficient funding, staff shortages, and low motivation hinder their effectiveness [104]. Particularly, these policies often overlook non-medical costs, posing a significant concern for adolescent women in LMICs [55,105]. Recommendations for improvement include enhancing drugs and laboratory services availability, increasing staff numbers, and improving funding for maternal healthcare programs. Furthermore, policies should address non-medical expenses, as solely focusing on medical costs may not effectively enhance maternal health service utilization in resource-constrained settings.

The access of adolescent women to maternal healthcare is influenced by service availability and acceptability. Factors such as proximity to facilities, long waiting times, and staff shortages affects access to these services. This is consistent with findings from previous studies done in South-East Asia and SSA [106,107]. Distant healthcare facilities pose challenges for adolescent women in accessing essential maternal health services. The shortage of healthcare professionals contributes to longer waiting times, reduced quality of care, and overall dissatisfaction with services [108]. Improving adolescent maternal healthcare in resource-limited settings, like SSA, requires enhancing the availability and accessibility of services. This involves expanding healthcare infrastructure, increasing the number of trained healthcare professionals, and ensuring comprehensive maternal healthcare for adolescent women.

### 4.1 Strength and limitation of the study

The strength of this review includes a comprehensive assessment of maternal health service utilization components and its barriers, thorough search strategies by including more than eight databases, and protocol registration on PROSPERO. In addition, we used Andersen's health-seeking model to comprehensively address the barriers of maternal health service utilization. The study has certain limitations. Firstly, the review focused on studies published only in English, potentially overlooking evidence, especially from Francophone Africa. The reliance on cross-sectional study designs in all quantitative studies limits causal inference. Moreover, the included studies represent only fifteen countries, potentially not capturing the full diversity of SSA countries. Lastly, the scarcity of relevant studies during the SDG era prevented comparison of pre- and post-SDG postnatal care utilization.

## 5 Conclusion and recommendations

In SSA, maternal health service utilization among adolescents remains at a low level. While there was a modest increase in ANC service utilization, the rise in SBA was more substantial from pre-SDG to the SDG era. Disparities across SSA regions and various barriers including predisposing, enabling, need and contextual barriers influence adolescent women maternal service utilization. These highlight the need to develop context-specific strategies and interventions targeting adolescent women. Addressing these challenges is crucial to achieving SDG3 by 2030.

## Ethical approval and consent to participate

Not applicable.

## Funding

This research did not receive any specific grant from funding agencies in the public, commercial, or not-for-profit sectors.

## Data availability

No data was used for this specific research article.

## CRediT authorship contribution statement

**Tadesse Tolossa:** Writing – review & editing, Writing – original draft, Visualization, Supervision, Software, Methodology, Funding acquisition, Formal analysis, Data curation, Conceptualization. **Lisa Gold:** Writing – review & editing, Writing – original draft, Visualization, Validation, Supervision, Resources, Project administration, Investigation, Formal analysis, Data curation, Conceptualization. **Merga Dheresa:** Writing – review & editing, Visualization, Validation, Supervision, Resources, Funding acquisition, Formal analysis. **Ebisa Turi:** Writing – review & editing, Visualization, Validation, Resources, Methodology, Formal analysis. **Yordanos Gizachew Yeshitila:** Writing – review & editing, Validation, Software, Resources, Investigation, Data curation. **Julie Abimanyi-Ochom:** Writing – review & editing, Writing – original draft, Validation, Software, Project administration, Investigation, Formal analysis, Data curation, Conceptualization.

## Declaration of competing interest

The authors declare that they have no known competing financial interests or personal relationships that could have appeared to influence the work reported in this paper.

## Supporting Information

S1 file**-**Database search results.

S2 file- JBI Quality assessment results.

## Abbreviation

ANC: Antenatal care
CI: Confidence Interval
OR: Odd Ratio
LMIC: Low- and Middle-Income Countries
MDG: Millennium Development Goal
PNC: Postnatal care
SBA: Skilled birth attendants
SSA: Sab-Saharan Africa
SDGs: Sustainable Development Goal
TBAs: Traditional birth attendants
WHO: World Health Organization
