# Supplementary material for: Adolescent maternal health services utilization and associated barriers in Sub-Saharan Africa: A comprehensive systematic review and meta-analysis before and during the sustainable development goals
Source: Heliyon. 2024 Aug 3;10(15):e35629. doi: 10.1016/j.heliyon.2024.e35629 (PMC11336889; doi:10.1016/j.heliyon.2024.e35629)
Supplement: Multimedia component 1 [file mmc1.docx]

**Table 1: Methodological qualities of qualitative studies**

| **Author** | **Is there congruity between the stated philosophical perspective and the research methodology?** | **Is there congruity between the research methodology and the research question or objectives?** | **Is there congruity between the research methodology and the methods used to collect data?** | **Is there congruity between the research methodology and the representation and analysis of data?** | **Is there congruity between the research methodology and the interpretation of results?** | **Is there a statement locating the researcher culturally or theoretically?** | **Is the influence of the researcher on the research, and vice- versa, addressed?** | **Are participants, and their voices, adequately represented?** | **Is the research ethical according to current criteria or, for recent studies, and is there evidence of ethical approval by an appropriate body?** | **Do the conclusions drawn in the research report flow from the analysis, or interpretation, of the data?** | **score** |  |
| --- | --- | --- | --- | --- | --- | --- | --- | --- | --- | --- | --- | --- |
|  |  |  |  |  |  |  |  |  |  |  |  |  |
| Apolot R et al. | Y | Y | Y | Y | Y | Y | Y | N | Y | N | 8 |  |
| Atuyambe L et al. | Y | Y | Y | N | N | N | Y | N | Y | Y | 6 |  |
| Hackett et al. | Y | Y | N | Y | N | Y | Y | Y | N | Y | 7 |  |
| Rukundo et al. | Y | Y | Y | Y | Y | Y | Y | N | Y | Y | 9 |  |
| Shatilwe et al. | Y | Y | N | Y | Y | Y | Y | N | N | Y | 7 |  |
| Samuel et al. | Y | Y | Y | N | Y | Y | Y | Y | Y | Y | 9 |  |
| Duggan et al. | Y | Y | Y | Y | N | N | Y | N | Y | Y | 7 |  |
| Mweteni et al. | Y | Y | Y | Y | Y | Y | Y | N | Y | Y | 10 |  |
| Chaibva et al. | N | Y | Y | N | Y | Y | Y | Y | Y | Y | 9 |  |
| Erasmus et al. | Y | Y | Y | N | Y | Y | N | Y | Y | Y | 8 |  |
| Bwalya et al. | Y | Y | Y | Y | N | Y | N | Y | Y | Y | 8 |  |
| Maria Ch. | Y | Y | Y | Y | N | Y | N | Y | Y | Y | 8 |  |
| Phafoli | N | Y | Y | N | Y | Y | Y | Y | Y | Y | 8 |  |
| Sewpaul et al | Y | Y | Y | Y | N | Y | N | Y | Y | Y | 6 |  |
| James S et al | Y | Y | Y | Y | N | N | Y | N | Y | Y | 7 |  |
| Chaibva et al. | Y | Y | Y | Y | N | N | Y | N | Y | Y | 7 |  |

**Table 2: Methodological qualities of quantitative studies**

| **Authors** | **Sample size** | **Were the criteria for inclusion in the sample clearly defined?** | **Were the study subjects and the setting described in detail?** | **Was the exposure measured in a valid and reliable way?** | **Were objective, standard criteria used for measurement of the condition?** | **Were confounding factors identified?** | **Were strategies to deal with confounding factors stated?** | **Were the outcomes measured in a valid and reliable way?** | **Was appropriate statistical analysis used?** | **Score** |
| --- | --- | --- | --- | --- | --- | --- | --- | --- | --- | --- |
| Tewdros A, et al. | 994 | Y | Y | Y | Y | N | Y | Y | Y | 7 |
| Rajesh K, et al. | 2434 | Y | Y | Y | Y | Y | N | N | Y | 6 |
| Olakunde B et al. | 789 | Y | Y | Y | Y | N | Y | N | Y | 6 |
| Onikepe O, et al. | 10052 | Y | Y | Y | Y | Y | N | Y | Y | 7 |
| Shamsu-Deen Ziblim | 120 | Y | Y | Y | N | N | N | Y | Y | 5 |
| Singh PK, et al. | 1,646 | Y | Y | N | Y | N | Y | Y | Y | 6 |
| Mekwunyi, et al. | 212 | Y | Y | N | Y | Y | Y | Y | Y | 6 |
| Akinyemi , et al.n Akinyemi | 3112 | Y | Y | Y | Y | Y | Y | N | Y | 7 |
| Rai RK, et al. | 934 | Y | Y | Y | Y | Y | Y | Y | Y | 8 |
| Thomas A, et al. | 898 | Y | Y | Y | Y | Y | Y | Y | Y | 8 |
| Mulunge N, et al. | 385 | Y | Y | Y | Y | Y | Y | Y | N | 7 |
| Grovogui F, et al. | 934 | Y | Y | Y | Y | Y | Y | N | Y | 7 |
| Rai Rk, et al. | 2160 | Y | Y | Y | Y | Y | Y | Y | Y | 8 |
| Atuyambe , et al. | 442 | Y | Y | Y | Y | Y | Y | Y | Y | 8 |
| C.A Alex, et al. | 2,096 | Y | Y | Y | Y | Y | Y | Y | Y | 8 |
| F. Iacoella, et al. | 4288 | Y | Y | Y | Y | Y | Y | Y | Y | 8 |
| C.A Alex, et al. | 4,775 | Y | Y | Y | Y | N | Y | Y | Y | 7 |
| Carvajal L, et al. | 22,135 | Y | Y | N | Y | Y | Y | N | Y | 6 |
| Banke T eta al. | 301 | Y | Y | N | Y | Y | Y | Y | Y | 7 |
| Govendera , et al. | 314 | Y | Y | Y | Y | Y | Y | Y | Y | 8 |
| Michael T et al | 621 | Y | Y | Y | Y | Y | Y | N | Y | 7 |


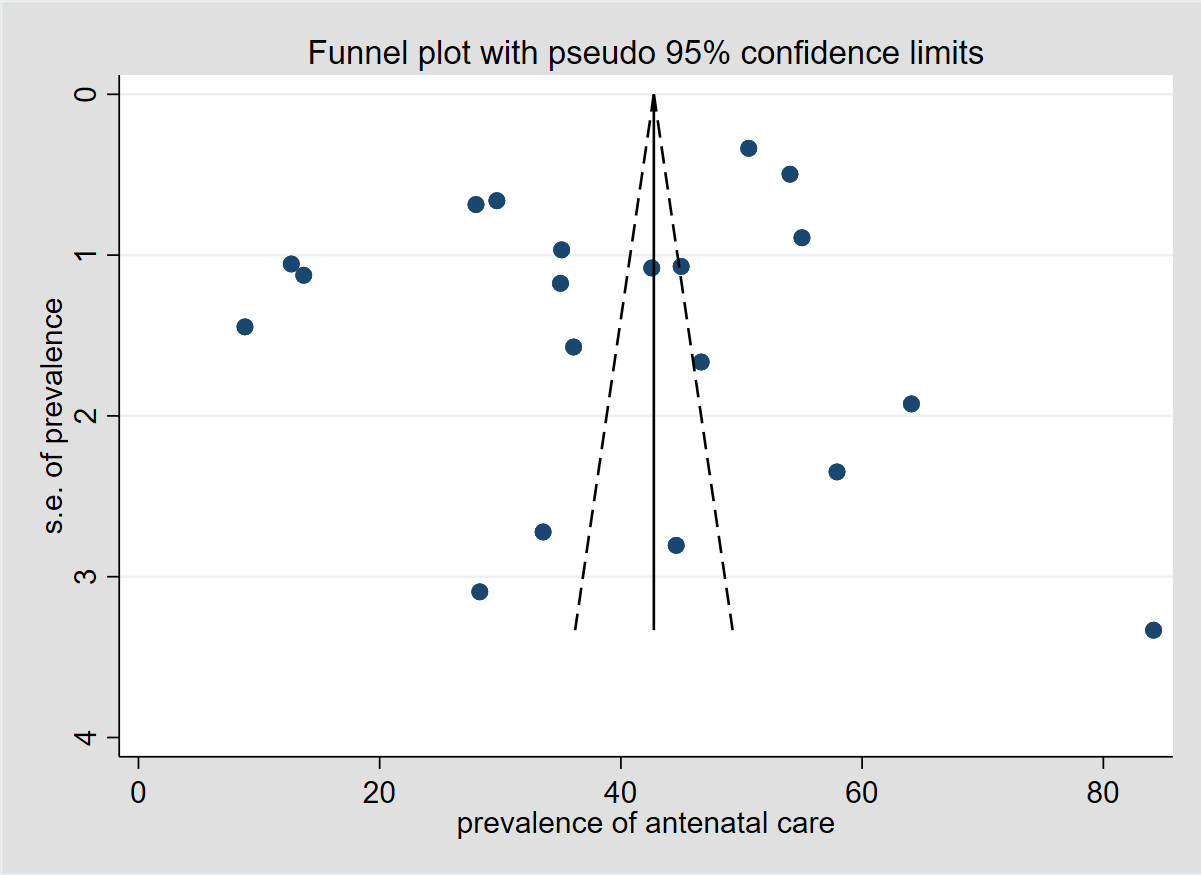


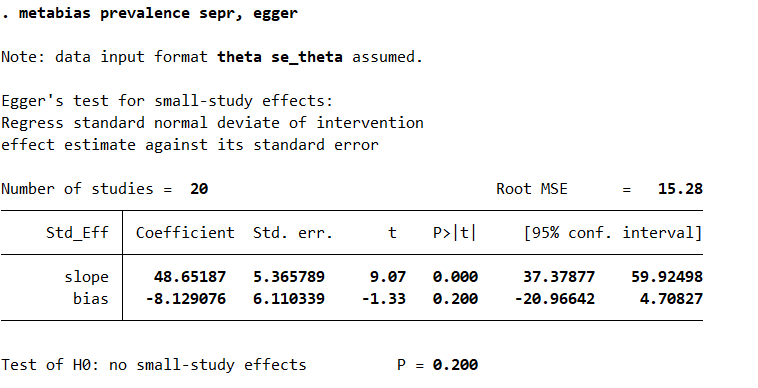


**Figure: Funnel plot with 95% confidence limits of the pooled level of ANC among adolescent women in SSA**


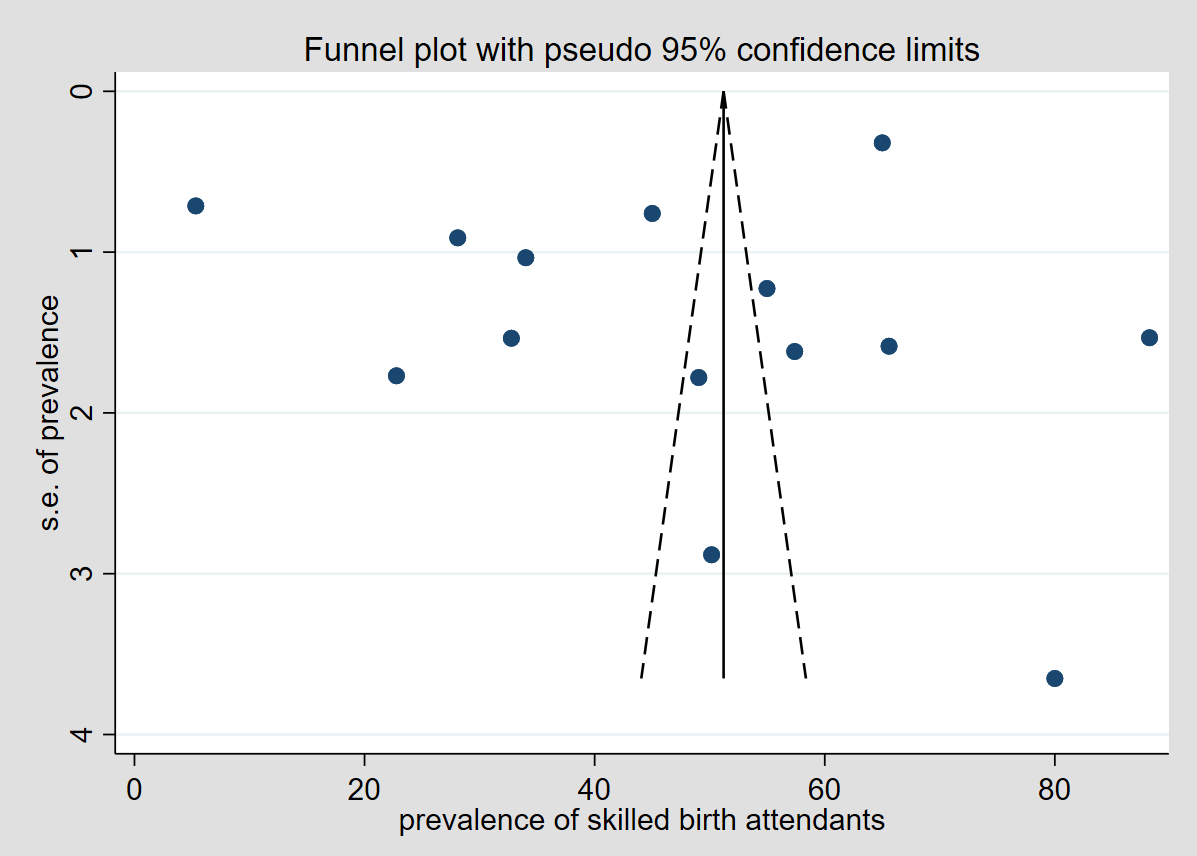


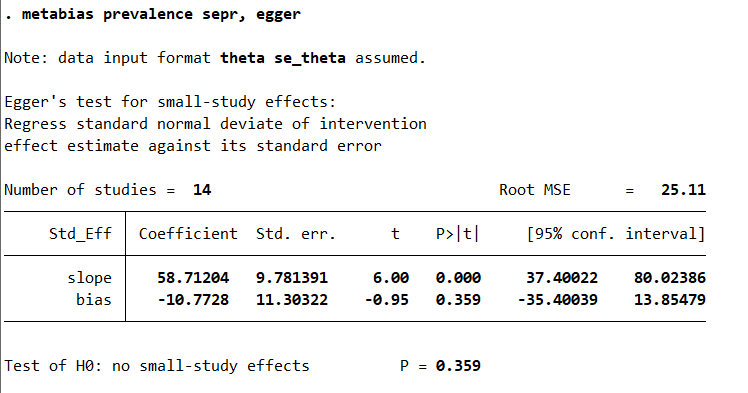


**Figure: Funnel plot with 95% confidence limits of the pooled level of SBA among adolescent women in SSA**
