# Supplementary material for: Adolescent maternal health services utilization and associated barriers in Sub-Saharan Africa: A comprehensive systematic review and meta-analysis before and during the sustainable development goals
Source: Heliyon. 2024 Aug 3;10(15):e35629. doi: 10.1016/j.heliyon.2024.e35629 (PMC11336889; doi:10.1016/j.heliyon.2024.e35629)
Supplement: Multimedia component 2 [file mmc2.docx]

**Database search result**

**1. PubMed search result**

| **S.No** | **Search terms** | **PubMed Search** | **#** |
| --- | --- | --- | --- |
| #1 | Maternal service utilization | **(((((((((((((((("maternal health"[MeSH Terms]) OR ("maternal health care")) OR ("maternal service utilization")) OR ("antenatal care")) OR (ANC)) OR ("prenatal care"[MeSH Terms])) OR ("institutional delivery")) OR ("skilled birth attendants")) OR ("home delivery")) OR ("postnatal care"[MeSH Terms])) ) OR ("postpartum care")) OR ("postnatal utilization")) OR ("maternal continuum of care")) OR ("obstetric care")) OR (EmOC)** | 57, 102 |
| #2 | Determinants | Determinants OR “associated factors” OR “risk factors” OR “social determinants” OR predictors | [2,251,654](https://pubmed.ncbi.nlm.nih.gov/?term=%28%28%28%28%28%28adolescen%2A%5BMeSH+Terms%5D%29+OR+%28%22adolescent+mothers%22%5BMeSH+Terms%5D%29%29+OR+%28%22adolescent+girls%22%29%29+OR+%28juvenile%29%29+OR+%28youth%2A%29%29+OR+%28%22young+women%22%29%29+OR+%28teenager%5BMeSH+Terms%5D%29+AND+%28humans%5BFilter%5D%29&sort=&size=200) |
| #3 | Adolescents | **((((((adolescen*[MeSH Terms]) OR ("adolescent mothers"[MeSH Terms])) OR ("adolescent girls")) OR (juvenile)) OR (youth*)) OR ("young women")) OR (teenager[MeSH Terms])** | 2,441,265 |
| #4 | SSA | "Sub-Saharan Africa" OR Angola OR Benin OR Botswana OR Burkina Faso OR Burundi OR Cameroon OR “Central African Republic” OR Chad OR Congo OR Comoros OR “Cote d'Ivoire” OR ”Democratic Republic of the Congo” OR “Equatorial Guinea” OR Eritrea OR Ethiopia OR Gabon OR Gambia OR Ghana OR “Guinea-Bissau” OR Kenya OR Lesotho OR Liberia OR Madagascar OR Malawi OR Mali OR Mauritania OR Mozambique OR Namibia OR Niger OR Nigeria OR “Republic of Congo” OR Rwanda OR “Sao Tome” OR Principe OR Senegal OR “Sierra Leone” OR Somalia OR “South Africa” OR Sudan OR Swaziland OR Tanzania OR Togo OR Uganda OR Zambia OR Zimbabwe | 487,055 |
| **#5** |  | **#1 AND #2 AND #3 AND #4**  Filters: **Humans, from 2000 - 2022** | **1836** |

**2. Medline search**

| **#** | **Query** | **Results from 29 Oct 2022** |
| --- | --- | --- |
| 1 | exp "Patient Acceptance of Health Care"/ or exp Health Services Accessibility/ or exp *Maternal Health Services/ or exp Pregnancy/ or maternal health [care.mp](http://care.mp/). or exp Maternal Health/ or exp Prenatal Care/ | 1,258,995 |
| 2 | prenatal [care.mp](http://care.mp/). or exp Prenatal Care/ | 37,856 |
| 3 | antenatal care.m_titl. | 2,322 |
| 4 | exp Pregnancy/ or ANC.mp. or exp Prenatal Care/ | 987,556 |
| 5 | skilled birth attendants.m_titl. | 88 |
| 6 | exp Midwifery/ or exp Prenatal Care/ or exp Health Services Accessibility/ or exp *Maternal Health Services/ or exp Adolescent/ or exp Pregnancy/ or skilled birth [attendants.mp](http://attendants.mp/). or exp Delivery, Obstetric/ | 3,203,302 |
| 7 | Home Childbirth/ or exp Pregnancy/ or exp Delivery, Obstetric/ or exp *Maternal Health Services/ or exp Health Services Accessibility/ or institutional [delivery.mp](http://delivery.mp/). or exp Adolescent/ | 3,196,478 |
| 8 | institutional delivery.m_titl. | 191 |
| 9 | home delivery.m_titl. | 227 |
| 10 | traditional birth [attendants.mp](http://attendants.mp/). or exp Midwifery/ | 21,430 |
| 11 | postnatal care.m_titl. | 452 |
| 12 | postpartum [care.mp](http://care.mp/). or exp Postnatal Care/ | 7,268 |
| 13 | Delivery, Obstetric/ | 32,482 |
| 14 | exp Delivery, Obstetric/ or exp *Maternal Health Services/ or exp Pregnancy/ or emergency obstetric [care.mp](http://care.mp/). or exp Health Services Accessibility/ | 1,108,742 |
| 15 | "adolescen*".m_titl. | 191,465 |
| 16 | "adolescen* women".m_titl. | 327 |
| 17 | "adolescen* females".m_titl. | 912 |
| 18 | exp Young Adult/ or exp *Adolescent/ or young [women.mp](http://women.mp/). | 1,022,996 |
| 19 | exp Pregnancy/ or exp *Adolescent/ or exp Pregnancy in Adolescence/ or teen*.mp. or exp Young Adult/ | 1,951,674 |
| 20 | determinants.m_titl. | 49,168 |
| 21 | associated [factors.mp](http://factors.mp/). | 30,636 |
| 22 | [predictors.mp](http://predictors.mp/). | 284,934 |
| 23 | Sub-Saharan Africa.mp. or exp *"Africa South of the Sahara"/ | 28,146 |
| 24 | exp Adolescent/ or exp Pregnancy/ or exp Delivery, Obstetric/ or exp Health Services Accessibility/ or exp *Maternal Health/ or exp *Maternal Health Services/ or maternal health service [utilization.mp](http://utilization.mp/). or exp Prenatal Care/ | 3,197,440 |
| 25 | exp Pregnancy/ or exp "Continuity of Patient Care"/ or maternal continuum of [care.mp](http://care.mp/). or exp Postnatal Care/ or exp *Maternal Health Services/ | 1,265,150 |
| 26 | "Delivery of Health Care"/ or socials determinants of [health.mp](http://health.mp/). | 111,284 |
| 27 | 1 or 2 or 3 or 4 or 5 or 6 or 7 or 8 or 9 or 11 or 12 or 13 or 14 or 24 or 25 | 3,571,220 |
| 28 | 15 or 16 or 17 or 18 or 19 | 2,122,827 |
| 29 | 20 or 21 or 22 or 26 | 467,998 |
| 30 | 23 and 27 and 28 and 29 | **724** |

**3. Embase search result**

| **No.** | **Query** | **Results** | **Date** |
| --- | --- | --- | --- |
| #32 | (('maternal care'/exp OR 'maternal care') OR 'maternal health service' OR 'prenatal care' OR 'antenatal care' OR anc OR 'skilled birth attendants' OR 'institutional delivery' OR 'home delivery' OR 'postnatal care'/exp OR 'early postnatal care' OR 'puerperium'/exp/mj OR 'continuum of care' OR 'obstetric procedure'/exp/mj OR 'puerperium'/exp) AND (determinants OR 'social determinants of health'/exp OR 'associated factors' OR predictors) AND ('adolescent women' OR adolescen* OR 'young adult'/exp OR 'adolescent girls' OR teenagers OR 'juvenile'/exp) AND ('africa south of the sahara'/exp OR 'sub-saharan africa' OR ssa) | **1795** | 28 Oct 2022 |
| #31 | 'africa south of the sahara'/exp OR 'sub-saharan africa' OR ssa | 329180 | 28 Oct 2022 |
| #30 | Ssa | 20561 | 28 Oct 2022 |
| #29 | 'sub-saharan africa' | 34315 | 28 Oct 2022 |
| #28 | 'africa south of the sahara'/exp | 302453 | 28 Oct 2022 |
| #27 | 'adolescent women' OR adolescen* OR 'young adult'/exp OR 'adolescent girls' OR teenagers OR 'juvenile'/exp | 4644177 | 28 Oct 2022 |
| #26 | 'juvenile'/exp | 4232806 | 28 Oct 2022 |
| #25 | teenagers | 19799 | 28 Oct 2022 |
| #24 | 'adolescent girls' | 11399 | 28 Oct 2022 |
| #23 | 'young adult'/exp | 478293 | 28 Oct 2022 |
| #22 | adolescen* | 2058853 | 28 Oct 2022 |
| #21 | 'adolescent women' | 1376 | 28 Oct 2022 |
| #20 | determinants OR 'social determinants of health'/exp OR 'associated factors' OR predictors | 693340 | 28 Oct 2022 |
| #19 | predictors | 437072 | 28 Oct 2022 |
| #18 | 'associated factors' | 37716 | 28 Oct 2022 |
| #17 | 'social determinants of health'/exp | 15440 | 28 Oct 2022 |
| #16 | determinants | 238281 | 28 Oct 2022 |
| #15 | ('maternal care'/exp OR 'maternal care') OR 'maternal health service' OR 'prenatal care' OR 'antenatal care' OR anc OR 'skilled birth attendants' OR 'institutional delivery' OR 'home delivery' OR 'postnatal care'/exp OR 'early postnatal care' OR 'puerperium'/exp/mj OR 'continuum of care' OR 'obstetric procedure'/exp/mj OR 'puerperium'/exp | 411687 | 28 Oct 2022 |
| #14 | 'puerperium'/exp | 80948 | 28 Oct 2022 |
| #13 | 'obstetric procedure'/exp/mj | 254879 | 28 Oct 2022 |
| #12 | 'continuum of care' | 4479 | 28 Oct 2022 |
| #11 | 'puerperium'/exp/mj | 23700 | 28 Oct 2022 |
| #10 | 'early postnatal care' | 55 | 28 Oct 2022 |
| #9 | 'postnatal care'/exp | 134058 | 28 Oct 2022 |
| #8 | 'home delivery' | 4971 | 28 Oct 2022 |
| #7 | 'institutional delivery' | 891 | 28 Oct 2022 |
| #6 | 'skilled birth attendants' | 593 | 28 Oct 2022 |
| #5 | anc | 13521 | 28 Oct 2022 |
| #4 | 'antenatal care' | 15291 | 28 Oct 2022 |
| #3 | 'prenatal care' | 53367 | 28 Oct 2022 |
| #2 | 'maternal health service' | 2792 | 28 Oct 2022 |
| #1 | 'maternal care'/exp OR 'maternal care' | 56806 | 28 Oct 2022 |

**4. Cinahl complete**

| **#** | **Query** | **Limiters/Expanders** | **Last Run Via** | **Results** |
| --- | --- | --- | --- | --- |
| S1 | maternal health or pregnancy or perinatal | Search modes - Boolean/Phrase | Interface - EBSCOhost Research Databases Search Screen - Advanced Search Database - CINAHL Complete | 296,652 |
| S2 | TI maternal service utilization | Search modes - Boolean/Phrase | Interface - EBSCOhost Research Databases Search Screen - Advanced Search Database - CINAHL Complete | 191 |
| S3 | TI maternal continuum of care | Search modes - Boolean/Phrase | Interface - EBSCOhost Research Databases Search Screen - Advanced Search Database - CINAHL Complete | 42 |
| S4 | TI prenatal care or pre-natal care or perinatal care | Search modes - Boolean/Phrase | Interface - EBSCOhost Research Databases Search Screen - Advanced Search Database - CINAHL Complete | 2,802 |
| S5 | TI Institutional delivery | Search modes - Boolean/Phrase | Interface - EBSCOhost Research Databases Search Screen - Advanced Search Database - CINAHL Complete | 150 |
| S6 | TI home delivery | Search modes - Boolean/Phrase | Interface - EBSCOhost Research Databases Search Screen - Advanced Search Database - CINAHL Complete | 415 |
| S7 | TI traditional birth attendants | Search modes - Boolean/Phrase | Interface - EBSCOhost Research Databases Search Screen - Advanced Search Database - CINAHL Complete | 221 |
| S8 | TX postnatal care or postpartum or early postnatal period or postnatal support or care after birth or care following birth | Search modes - Boolean/Phrase | Interface - EBSCOhost Research Databases Search Screen - Advanced Search Database - CINAHL Complete | 60,795 |
| S9 | TX obstetric care | Search modes - Boolean/Phrase | Interface - EBSCOhost Research Databases Search Screen - Advanced Search Database - CINAHL Complete | 65,361 |
| S10 | TX emergency obstetric care | Search modes - Boolean/Phrase | Interface - EBSCOhost Research Databases Search Screen - Advanced Search Database - CINAHL Complete | 1,778 |
| S11 | S1 OR S2 OR S3 OR S4 OR S5 OR S6 OR S7 OR S8 OR S9 OR S10 | Search modes - Boolean/Phrase | Interface - EBSCOhost Research Databases Search Screen - Advanced Search Database - CINAHL Complete | 349,422 |
| S12 | TI determinants | Search modes - Boolean/Phrase | Interface - EBSCOhost Research Databases Search Screen - Advanced Search Database - CINAHL Complete | 18,047 |
| S13 | TX social determinants of health | Search modes - Boolean/Phrase | Interface - EBSCOhost Research Databases Search Screen - Advanced Search Database - CINAHL Complete | 28,723 |
| S14 | TI associated factors | Search modes - Boolean/Phrase | Interface - EBSCOhost Research Databases Search Screen - Advanced Search Database - CINAHL Complete | 25,562 |
| S15 | TI predictors or risk factors or causes or predisposition or determinants or cause | Search modes - Boolean/Phrase | Interface - EBSCOhost Research Databases Search Screen - Advanced Search Database - CINAHL Complete | 155,010 |
| S16 | S11 S12 OR S13 OR S14 OR S1 | Search modes - Boolean/Phrase | Interface - EBSCOhost Research Databases Search Screen - Advanced Search Database - CINAHL Complete | 200,264 |
| S17 | TI adolescents or teenagers or young adults | Search modes - Boolean/Phrase | Interface - EBSCOhost Research Databases Search Screen - Advanced Search Database - CINAHL Complete | 117,629 |
| S18 | TI young women or young females or young adults | Search modes - Boolean/Phrase | Interface - EBSCOhost Research Databases Search Screen - Advanced Search Database - CINAHL Complete | 27,947 |
| S19 | S18 OR S19 | Search modes - Boolean/Phrase | Interface - EBSCOhost Research Databases Search Screen - Advanced Search Database - CINAHL Complete | 124,436 |
| S20 | TX sub saharan africa or sub-saharan africa or sub sahara or sub-sahara or ssa | Search modes - Boolean/Phrase | Interface - EBSCOhost Research Databases Search Screen - Advanced Search Database - CINAHL Complete | 24,643 |
| S21 | TX S12 AND TX S17 AND TX S20 AND TX S20 | Search modes - Boolean/Phrase | Interface - EBSCOhost Research Databases Search Screen - Advanced Search Database - CINAHL Complete | 758 |
| S22 | TX S12 AND TX S17 AND TX S20 AND TX S21 | Search modes - Boolean/Phrase | Interface - EBSCOhost Research Databases Search Screen - Advanced Search Database - CINAHL Complete | **66** |

| **5. Scopus search result** | | |
| --- | --- | --- |
| 1 | ( TITLE-ABS-KEY ( "maternal service utilization" )  OR  TITLE-ABS-KEY ( "maternal healthcare" )  OR  TITLE-ABS-KEY ( "antenatal care" )  OR  TITLE-ABS-KEY ( anc )  OR  TITLE-ABS-KEY ( "institutional delivery" )  OR  TITLE-ABS-KEY ( "skilled birth attendants" )  OR  TITLE-ABS-KEY ( "home delivery" )  OR  TITLE-ABS-KEY ( "postnatal care" )  OR  TITLE-ABS-KEY ( "early postnatal care" )  OR  TITLE-ABS-KEY ( "maternal continuum of care" )  AND  TITLE-ABS-KEY ( determinants )  OR  TITLE-ABS-KEY ( "social determinants of health" )  OR  TITLE-ABS-KEY ( "associated factors" )  OR  TITLE-ABS-KEY ( predictors )  AND  TITLE-ABS-KEY ( adolescent* )  OR  TITLE-ABS-KEY ( "young women" )  OR  TITLE-ABS-KEY ( teen* )  OR  TITLE-ABS-KEY ( juvenile )  AND  TITLE-ABS-KEY ( "Sub-Saharan Africa" )  OR  TITLE-ABS-KEY ( ssa ) ) | [**107**](https://www.scopus.com/search/history/results.uri?origin=searchhistory&shid=9) |

**6. APA Psycinfo VIA EBSCOhost**

|  | Search strategy | Number of records |
| --- | --- | --- |
| Maternal service utilization and its determinants among adolescent girls in SSA | **Search Alert:** "( 'maternal health care' OR 'maternal service utilization' OR 'antenatal care' OR ANC OR 'prenatal care' OR 'skilled birth attendance' OR 'institutional delivery' OR 'home delivery' OR 'postnatal care' OR 'early postnatal care' OR 'continuum of care' OR ‘obstetric care’ ) AND ( determinants OR ‘associated factors’ OR ‘risk factors’ OR 'social determinants of health' ) AND ( 'adolescen*' OR 'adolescent women' OR 'adolescent girls' OR juvenile OR 'adolescent women' OR youth ) AND ( "Sub-Saharan Africa" OR Angola OR Benin OR Botswana OR Burkina Faso OR Burundi OR Cameroon OR “Central African Republic” OR Chad OR Congo OR Comoros OR “Cote d'Ivoire” OR ”Democratic Republic of the Congo” OR “Equatorial Guinea” OR Eritrea OR Ethiopia OR Gabon OR Gambia OR Ghana OR “Guinea-Bissau” OR Kenya OR Lesotho OR Liberia OR Madagascar OR Malawi OR Mali OR Mauritania OR Mozambique OR Namibia OR Niger OR Nigeria OR “Republic of Congo” OR Rwanda OR “Sao Tome” OR Principe OR Senegal OR “Sierra Leone” OR Somalia OR “South Africa” OR Sudan OR Swaziland OR Tanzania OR Togo OR Uganda OR Zambia OR Zimbabwe ) Publication Year: 2000-2022 on 2022-10-24 11:46 PM" | **97**  **(1 dissertation)** |
